# Exploring postnatal and newborn home care experiences of rural and remote mothers in Pakistan whose neonates were admitted to the hospital: A qualitative study

**DOI:** 10.1371/journal.pone.0339957

**Published:** 2026-01-23

**Authors:** Mika Kuriyama, Moe Nakazora, Md Moshiur Rahman, Mari Sato, Saima Gillani, Sadia Habib, Yoko Shimpuku

**Affiliations:** 1 Graduate School of Biomedical and Health Sciences, Hiroshima University, Hiroshima, Japan; 2 Japan International Cooperation Agency, Tokyo, Japan; 3 Institute of Humanities and Social Sciences, University of Tsukuba, Tsukuba, Japan; 4 Tac International Inc., Tokyo, Japan; 5 Department of Pediatrics, Ayub Medical College, Abbottabad, Pakistan; 6 Department of Obstetrics and Gynecology, Ayub Medical College, Abbottabad, Pakistan; Menzies School of Health Research: Charles Darwin University, AUSTRALIA

## Abstract

**Background:**

The neonatal mortality rate in Pakistan has been the second highest worldwide. Since mortality rate is higher in rural area than in urban area, to understand newborn care and response to progressing disease by rural families is vital to comprehend the background of poor neonatal outcomes. But in Pakistan research on newborn home care is scarce. This study aimed to understand the local context of neonatal morbidity derived from rural and remote Pakistan by exploring the postnatal and newborn home care experiences of mothers whose neonates required admission after being cared for at home.

**Methods:**

This qualitative descriptive study conducted in-depth, individual, semi-structured interviews with 15 mothers and three family members with children admitted to the neonatal unit of a tertiary hospital from home. Seven pediatric and obstetric staff were also interviewed for data triangulation. Thematic analysis was used to extract themes and sub-themes, and to develop a thematic map.

**Results:**

Lack of early initiation and exclusive breastfeeding, administration of prelacteals, and suboptimal bathing, wiping, wrapping, and umbilical cord care were identified; they were influenced by family experiences. Short postnatal stays, and lack of postnatal care and health education from facilities, left mothers with traditional care only. Very low birth weight neonates had been cared for at home. From the analysis, five themes were identified. They were gender roles, health literacy, health seeking behavior, lack of preventive care, and a high burden of low birth weight.

**Conclusion:**

This study identified local newborn home care influenced by familial traditional norms that could make neonates susceptible to infection, dehydration, malnutrition, and hypothermia. Multiple healing approaches and health service quality may have affected families’ ability to seek timely medical attention. Strengthening facility preventive care, and families’ health literacy through culture-sensitive health education, is essential in antenatal and postnatal care, and during admission. Appropriate timing of home discharge, with newborn care counselling, is also vital, considering the high burden of preterm births and low birth weight.

## Introduction

The neonatal mortality rate in Pakistan was 41 in 2019 [1] and 39 in 2021–2022 [2,3], per thousand live births, which has been the second highest in the world. Causes of neonatal death in Pakistan have not changed for almost two decades; preterm birth accounts for 36.1%, followed by intrapartum complications at 22.1%, sepsis at 18.6%, pneumonia at 6.3%, and congenital anomalies at 6.2% [4]. They are similar to global trends identified by the World Health Organization (WHO); premature birth, birth complications such as birth asphyxia and trauma, neonatal infections, and congenital anomalies remain the leading causes of neonatal deaths [5].

The background of high neonatal mortality in Pakistan is complex. In a study comparing low and middle-income countries, Pakistan had the poorest pregnancy outcomes in terms of maternal and neonatal mortality, stillbirth, preterm birth, and low birth weight (LBW). The suggested reasons included a low level of education, malnutrition and anemia in women of reproductive age, and inadequate care at the site of preterm and LBW delivery [6]. Pakistan has one of the highest global LBW burdens; it was as high as 22% in 2017–2018 [7], although most newborns (89%) are not weighed at birth [3].

Newborn care is critical for neonatal survival. Since neonatal mortality rate is higher in rural area than in urban area [7], to understand newborn care by rural families is vital to comprehend the background of poor neonatal outcomes. In particular, caregivers’ narratives of how families cared for newborns at home, how newborn illnesses progressed, how caretakers sought medical attention, how mothers and newborns attended postnatal care (PNC), and what care and health education mothers received, could help us understand the phenomenon better. In Pakistan, there has been limited qualitative research on the topic, with study areas clustered geographically [8,9]. Therefore, it is difficult to clearly understand how newborns are cared for at home in rural and remote areas.

A study examining knowledge, attitudes, and practice regarding home care of newborns in Sindh Province, Pakistan, found that nearly half bathed their newborns within six hours, did not feed colostrum, and administered prelacteals (non-breastmilk substances administered to the newborn during the first three days) including animal and formula milk, honey, and *ghee* (clarified butter), which suggests that WHO recommended newborn care is not practiced [8]. The early initiation of breastfeeding rate, within one hour of delivery, in Pakistan is unreasonably low at 15.4%, compared with 80.9% in India [6]; this raises the concern that the importance of breastfeeding is poorly recognized at home and at birth facilities. Additionally, PNC issues and lack of counseling have been identified. In a survey on the quality of PNC in an urban hospital in Pakistan, maternal and neonatal examination was not done for 30% to 50% of patients and information regarding danger signs was not provided in 69% of cases [10]; this leaves room for improvement of postnatal health service delivery. Nevertheless, the reasons for suboptimal newborn care action by families are not clearly understood, and newborn home care practices in other areas of Pakistan have not been researched yet.

This study aimed to understand the local context of neonatal morbidity derived from rural and remote Pakistan. The unique aspect of this study is that we qualitatively explored the experiences of the mothers and families who cared their neonates at home, and their neonates actually fell sick and were brought to the hospital from rural community. It would allow us to uncover the experiences which could be the potential contributors of their newborn ill state. This study chose a target site in Khyber Pakhtunkhwa (KP) Province. This is one of the provinces that has lagged in development due to the influx of Afghan refugees, mountainous terrain, and natural disasters. Reducing neonatal mortality is an overriding priority of the provincial health department, but antenatal care (ANC) utilization and facility delivery remain lower than the national average [11]. Local tribal customs are considered to impact on women’s access to healthcare services, in addition to geographical and economic factors. By exploring newborn home care, especially that in rural and remote areas in the region, this study can provide valuable insights for healthcare staff, health administration, policymakers, and development partners about newborn home care practices in reality to enable the necessary support with priorities for newborns, mothers, and their families in rural Pakistan.

## Methods

### Setting

**Fig 1:**
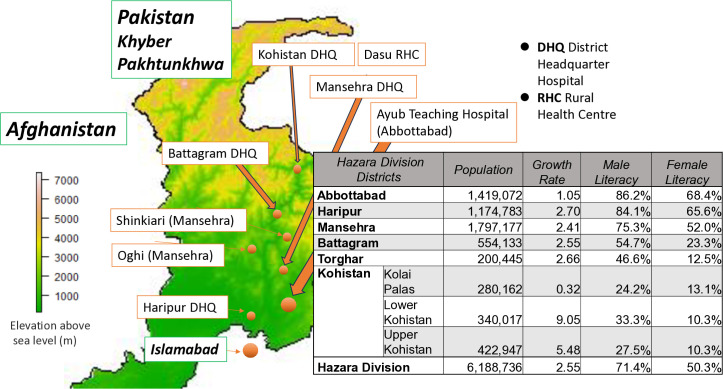
Geographic location and district profiles; Map and table generated using GADM data (version 2.8) for Pakistan, and Pakistan Bureau of Statistics Census 2023 data, accessed December 2025 (Source: gadm.org; census23.pbos.gov.pk/).

This study was conducted at Ayub Teaching Hospital (ATH) in Abbottabad, a 1460-bed hospital that is the largest and only tertiary healthcare facility in Northern Pakistan, providing services to millions of people living in and around Hazara division and the surrounding territory [12]. The total population of the Hazara division of KP province is 6,188,736, in a total area of 15,649 km^2^. Fig 1 shows geographical location and district profile of Hazara division [13,14]. The major linguistic and ethnic groups include Hindkowans, Pashtuns, and Kohistanis [15]. With its vast catchment area and professional availability, the facility has been the last resort for severely ill patients in the region. Therefore, it is ideal for studying patients from peripheral areas who require advanced treatment.

### Design

A qualitative descriptive study design was used. Data were collected through in-depth individual interviews with mothers or family members whose neonatal children were admitted to the hospital. In qualitative research, the use of triangulation, and multiple methods or data sources, can provide a more comprehensive understanding of the phenomenon under study [16]; therefore, we also sourced data from hospital staff, and enhanced it with observations of clinical and surrounding areas in the hospital.

### Participants

Purposeful sampling was used to identify participants that could serve the study purpose. The inclusion criteria for mothers and family members were: (1) their child was admitted to the newborn care unit after being cared for at home, (2) they came or were referred from a rural/remote area from Mansehra, Battagram, Torghar, and Kohistan Districts which were considered as more remote areas in Hazara division, compared to the rest of two Districts, (3) they understood Urdu and/or Pashtun, and (4) they agreed to participate in the study. Since the aim of this study is to understand the local and remote context, residents from Abbottabad and Haripur Districts in Hazara division were excluded, due to relatively easier access to urban hospitals in the two Districts and Islamabad. Hospital staff from the neonatal unit and the obstetric department were included for data triangulation.

### Recruitment

Recruitment and interviews were conducted from January to March 2024. Each morning, on interview dates, the head nurses of relevant clinical areas were contacted to identify eligible potential participants based on the inclusion criteria. Since families were not allowed to stay in the neonatal unit, they attended to the unit only when they were called from the hospital staff to bring formula or extracted breastmilk and diapers, or for the briefing of patient situation. When families from the targeted areas presented, the head nurse called the research team, then the team approached and briefed about the research. Mothers were priority, but it was revealed that there were not many postnatal mothers due to the harsh environment for attendants, so the team included the family members who could narrate how the newborn was delivered, being cared at home, developed symptoms and brought to the hospital. The research team included the first author who is a non-Pakistani female with a nursing background in Australia and Japan and three years of experience in Pakistan, and a female Pakistani non-clinical interpreter, fluent in Pashtun, Urdu, and English.

### Data collection and tool

A semi-structured interview guide was created in English by the first author, and cross checked by the last author (S1 Appendix). A quiet meeting room or a staff room was allocated as a venue to maintain privacy. The first author, with the interpreter, conducted interviews in Urdu, Pashtu, and English. Interviews were audio-recorded and field notes were taken after obtaining consent. Participants were not accompanied by their relatives, other than two mothers from Kohistan who only spoke Kohistani so their female relative interpreted from Kohistani to Pashtu or Urdu. Each interview took 30 minutes on average. The newborns’ medical record was cross-checked to understand their disease progression including diagnosis and weight on each interview day (11th and 17th of January, 22^nd^, 26 and 29^th^ of February, 4^th^ 7^th^ and 11^th^ of March, 2024). The interviews were conducted until saturation, the point at which no new relevant information is emerging. After the data collection, the participants’ information that directly links data to an individual was de-identified for data analysis. The audio-recorded interviews were transcribed into English verbatim by the same interpreter, with the aid of field notes.

### Data analysis

Qualitative thematic analysis, as described by Braun and Clarke [17], was used to guide the analysis. The first author repeatedly read the transcripts to get immersed in the worldview narrated by the participants, searching for meaning and patterns of perception, behavior, and environment that could have contributed to, or prevented, the illness state of the neonates. Meaning units were extracted from transcripts and initial coding was generated manually. Then similar codes were grouped into sub-themes, and themes were created through abstraction. The table with candidate themes, sub-themes, codes, and relevant quotes was developed by the first author, and the other authors reviewed them for refinement. Based on the refined and reorganized table, a thematic map showing the relationship between the themes, sub-themes, and codes was developed by the first author, followed by discussion with the research team for final agreement.

### Ethics

Ethical reviews and approvals were obtained from: (1) the institutional ethical review committee at Ayub Medical & Teaching Institution, Abbottabad, Pakistan (Ref.No.RC-EA-2023/122–8011) and (2) the research ethics review board at Hiroshima University, Hiroshima, Japan (E2023-0197). During recruitment, the participants were briefed about the study objective, procedures, and measures to ensure privacy and anonymity. The participants were informed that they could withdraw at any time and it would not affect the relationship with the hospital and the therapeutic team. After the briefing, each participant provided written informed consent to participate in the study voluntarily and to be recorded with an audio-recorder.

## Results

A total of 15 mothers, three family members, and seven hospital staff were interviewed. The mothers’ ages ranged from 18 to 45 years old. Of the 18 families including 15 mothers and three family members, nine resided in Mansehra, six in Battagram, and three in Kohistan, including Upper Kohistan and Kolai Palas district (Table 1).

**Table 1:** Demographic data.

ID	Relation to neonate	Native language	Residence	Mother age	Mother education	Father job	Maternal age at marriage	Family living with	Obstetric History	Neonate Admitted at	Interviewed at
1	Mother	Kohistan	Kohistan	21	Bachelor	Driver	17	28	G5P3(3 miscarried)	Day 22	Day 22
2	Mother	Hindko	Mansehra	45	No education	Daily labor	25	8 (Nuclear)	G12P5(7 miscarried)	Day 10?	Day 10?
3	Mother	Hindko	Battagram	24	No education	Army	20	12	2G2P(1 died at 3M)	Day 9	Day 15
4	Mother	Kohistan	Kohistan	30	No education	Teacher	13	20	6G6P(2 died)	Day 8	Day 10
5	Mother	Kohistan	Mansehra	29	Grade 12	Carpenter	25	20	3G3P	Day2	Day 4
6	Mother	Hindko	Mansehra	26	No education	Snack restaurant	14	27	G8P6(1miscarried 2died)	Day 27	Day 28
7	Mother	Pashtun	Battagram	22	Grade 5	Daily labor	14	4 (Nuclear)	G2P2	Day 17	Day 17
8	Mother	Hindko	Mansehra	32	Grade 12	Daily labor	25	5(Joint)	G6P4(2miscarried 2died)	Day 17	Day 23
9	Mother	Hindko	Mansehra	39	Grade 8	Restaurant	26	39	G4P3(1miscarried)	Unknown	Day 12
10	Mother	Hindko	Mansehra	23	Grade 12	Farmer	19	4 (Nuclear)	G2P2(Twin died at Day19)	Day 20-22	Day 30
11	Mother	Pashtun	Battagram	25	Grade 8	Not working	21	4(Joint)	G2P1(1 miscarried)	Day 12	Day 13
12	Mother	Pashtun	Kohistan	29	No education	Policeman	17	23	G5P5	Unknown	Day 26
13	Mother	Pashtun	Battagram	18	No education	Student	17	20	G1P1	Day 19	Day 22
14	Mother	Hindko	Mansehra	20	Bachelor	General Store	18	5(Joint)	G2P2(1died at Day1)	Day 2-3	Day 4
15	Mother	Hindko	Mansehra	20	Grade 8	Livestock	18	7(Joint)	G2P2	Day 12	Day 40
16	Father	Pashtun	Battagram	30	Grade 10	Daily labor	18	7(Nuclear)	G7P7(2died)	Day 2	Day 3
17	Aunt	Hindko	Mansehra	22	Grade 10	Driver	?	15	G5P4 (1 miscarried)	Unknown	Day 6
18	Grandfather	Gojri	Battagram	25	No education	Work in Karachi	23	14	G2P1(1miscarried)	After Day7	Day18
ID	Profession	Language	Workplace	Age	Work experience						
19	Doctor	Hindko	Newborn	33	4 years						
20	Doctor	Pashtun	Newborn	47	18 years						
21	Nurse	Hindko	Newborn	45	19 years						
22	Nurse	Hindko	Obstetrics	46	20 years						
23	Nurse	Chitorali	Obstetrics	48	23 years						
24	Nurse	Hindko	Obstetrics	29	8 years						
25	Nurse	Hindko	Newborn	48	25 years						

Common neonatal medical issues were preterm and/or LBW (7), jaundice (4), and pneumonia (4). Newborn weight ranged from 1.1 kg to 3.4 kg. Only one mother initiated breastfeeding within one hour of birth, and most of the newborns were given prelacteals such as *kahwa* (tea), animal milk, and infant drops. Three mothers stated that they never bathed or wiped the baby at home (ages Day 4, Day 4, and Day 28 on the interview date). Ten caregivers out of the 18 applied agents on the neonate’s umbilical cord, including mustard oil, spirits, ointment, and *kajal/kohl/surma* (blackish eye cosmetic). Only four of the 18 newborns were given zero dose routine immunization (Table 2).

**Table 2:** Newborn diagnosis and care.

ID	Rederral/ Delivery Place	Neonate diagnosis	Neonate weight	BF initation	Prelacteals	Bath/Wipe	Cord care	ANC	PNC	Zero dose
1	Dasu RHC-Private-ATH	(Cervical cerclage), Pneumonia	3.0 kg	Unknown	Infant drops	Unknown	Unknown	8 Private	No	No
2	Abbottabad Private	Preterm (8.5M), LBW, NNJ, Sepsis	2.3 kg	Unknown	Unknown	Unknown	Unknown	7 Private	Mother	Yes
3	Battagram DHQ	NNJ, Meningitis?	Unknown	Day 3	Formula	Unknown	Unknown	3 Private	No	No
4	Battagram DHQ	Fits, Diarrhea	3.0 kg	2 hours	Tea, Cowmilk, Water, Formula	No bath/Wipe Day1	None	Monthly Govt	No	No
5	Abbottabad Private	NNJ, Sepsis	3.2 kg	Day 2	Formula	No Bath/No Wipe	Spirit	? times Private	Unknown	No
6	Oghi Private	Prolonged vomit, LBW (9M twins)	1.1 kg	Day 2	Tea, Formula	No Bath/No Wipe	Ointment	Monthly Private	Unknown	No
7	ATH	Pneumonia	Unknown	10 minutes	Tea for 3 days	No bath/Wiped	Kajal/Kohl	3 ATH	Unknown	No
8	Garhi Habibullah Private	Fits, Hypocalcemia	3.0 kg	Day 3	Infant drops	Bath Day6	Spirit, Mustard oil	2 ATH, 5 Private	No	Yes
9	Mansehra Home	NNJ, Anemia	2.5 kg	Day 2	Goat milk	Bath Day0	Mustard oil	4 Private, 1 DAI	Unknown	Yes
10	Mansehra DHQ-ATH	Preterm (8M), LBW	1.5 kg	Day 12	Formula	No bath at home	(in Nursery Day0–12)	2 Private, 7 DHQ	No	No
11	Battagram Private	Anemia, Preterm (8M10D), LBW	1.7 kg	Day 3	Formula	Bath Day8	Mustard oil, Powder	3 Private	Unknown	Unknown
12	Oghi Private	Pneumonia	3.4 kg	Day 3	Infant drops	No bath/Wipe Day2	Mustard oil	4-5 Private	No	No
13	Government-ATH	Pneumonia, NNJ	“Normal”	Day 4	Formula	No bath/Wipe Day4	Mustard oil	3 Govt	Unknown	Yes
14	Shinkiari Private	Preterm(8M), Sepsis	2.5 kg	Day 3	Tea, Dates	Never at home	Unknown	Monthly private	Mother	No
15	In Ambulance to ATH	Preterm (7.5M), LBW, Anemia	1.6 kg	Never	Formula	No bath/Wiped	Mustard oil, ointment	5 Private,2 Govt	No	No
16	Battagram Private	Fever, Fits, HIE	3.0 kg	3-4hrs	Tea	No bath/Wipe Day2	Unknown	2 Private, 3 DHQ	Unknown	No
17	Shinkiari Private	LBW, Swollen abdomen	2.0 kg	Never	Formula	No bath/Wiped	Ointment	Monthly Private	Mother	No
18	Battagram Home	HIE, TGA	2.8 kg	2-3hrs	None	No bath/Wiped	Unknown	3 Govt	Unknown	No

*BF: Breastfeeding, ANC: Antenatal Care, PNC: Postnatal Care, RHC: Rural Health Center, ATH: Ayub Teaching Hospital, DHQ: District Headquarter Hospital, Govt: Government (Health Facility), LBW: Low Birth Weight, NNJ: Neonatal Jaundice, Kajal/Kohl: Blakish eye cosmetic, HIE: Hypoxic Ischemic Encephalopathy, TGA:Transposition of the great arteries.*

Three individual/family-related themes were generated from the interviews and subsequent analysis. Theme one was gender roles inside and outside the home. Theme two discusses health literacy, influenced by family experiences. Theme three describes health seeking behavior, influenced by family circumstances. One institution-related theme was abstracted as a lack of preventive care. A general, overarching theme was extracted as the high burden of LBW (Table 3).

**Table 3:** Themes and subthemes.

*Themes*	*Subthemes*
**Gender roles**, inside and outside the home	Female role inside the home to bear and rear children within the family structure
	Male decision making outside the home without involvement in women’s business
**Health literacy**, influenced by family experiences	Lack of early initiation and exclusive breastfeeding
	Suboptimal bathing, wiping, wrapping and umbilical cord care
	Influence by local/traditional family experiences
**Health seeking behavior**, influenced by family circumstances	Home treatment by family knowledge and experiences
	Customs to seek *moluvi (*local Islamic preacher)
	Distrust/quality in rural health facilities
Lack of **preventive care**	No reminder of regular PNC and immunization
	Short duration of postnatal stay in health facility
	Limited/ineffective health education/counselling
High burden of **LBW**	Maternal malnutrition
	Poor understanding of complications
	LBW and VLBW cared for at home

**Fig 2:**
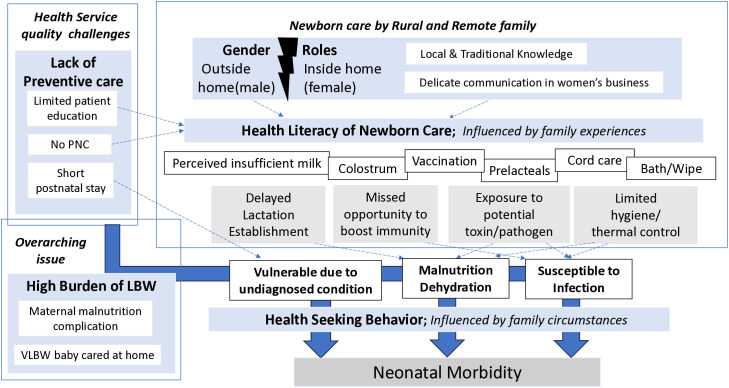
Thematic map.

A thematic map was created to illustrate how those themes and sub-themes related to each other, and how they could lead to newborn morbidity (Fig 2).

### Theme 1: gender roles inside and outside the home

Gender roles were explicitly divided inside and outside the home. Whilst pregnancy, childbirth, and infant care were the responsibility of females, decision making regarding travel and finance was a male responsibility.

#### a. Female role inside the home to bear and raise children within the familial structure.

In most cases, the mother was the primary caregiver to nurse the newborn; therefore, she noted deviations from her neonate’s normal state, including breathing difficulties, skin color change, vomiting, fever, fits, body swelling, feeding challenges, or over-crying.

*“She was all fine and we went back home after the delivery. It’s been three days since the baby had not been feeling well (from Day 14). She could not drink milk. She had shortness of breath. There was an unusual voice coming out from her nose. She also had a thick stool. Some people said she had pneumonia, so we went to the hospital. She has a chest infection and abdominal issues. Also, she got a severe temperature. My husband said we go to ATH. It’s a good hospital.”* (Mother, Battagram, 7)

Along with the local *purdah* (women seclusion) custom, females typically stayed at home, responsible for house chores and childbearing, without much focus on education.

*“[Are you working or housewife?] Housewife. We are Pashtuns, we don’t go outside home*”. (Mother, Battagram, 7)

*“I went to school for 15 days only. My brothers were strict saying we are Syeds (descendants of the Islamic prophet Muhammad, expected to be Muslim role model families), didn’t allow me after that. But I really wanted to study and (remembered) little bit of words I learned. Now I have kids and send them to read (She was married at 14).”* (Mother, Mansehra, 6)

Daughters-in-law were expected to have male children, reflecting the local belief that boys are the heirs of the family and land, and become breadwinners to help with their kin-group finance, whilst girls will leave the family on marriage, with parents’ carrying the burden of dowry payment (bride payment) on their daughters’ marriage.

*“My brother-in-law died so my in-laws wanted to have a grandson as soon as possible. He died after 10 days of my daughter’s delivery and death. So, they said do not take any gap and conceive within that month.”* (Mother, Mansehra,14)

Infertility could threaten the position of the daughter-in-law in a familial structure; a staff member shared a story of a desperate woman who stole a baby from the hospital.

*“A woman came to kidnap a baby. We still have CCTV cameras in the nursery. She came with a handbag and shawl in it, to wrap the baby. There was no strictness in the nursery at that time. She wrapped the baby and was about to leave. She was captured at the last moment. Later on, she started saying, it’s been so many years of my marriage and I don’t have kids. My husband will divorce me and he will do this and that....”* (Staff, 25)

#### b. Male decision making outside the home without involvement in women’s business.

Male figures in families were not directly involved in newborn care at home; however, they were responsible for decisions to seek medical attention outside. Women are required to be accompanied by male family members when it is necessary to go out.

*“The baby was delivered at night. I was discharged next morning. They kept the baby in a glass (incubator) for three days. Then we spent nine days at home. And last night we came to ATH. The baby turned yellow as she had jaundice. We placed the baby in the sunlight and the skin turned extremely yellow. My husband decided to go to the hospital. He came back from Lahore the next day he was born. We took local transport. My husband and his brother accompanied, and we stay outside in the hospital.”* (Mother, Battagram, 11)

Male members were also expected to provide money for transport and hospital expenses for patients and attendants to stay; however, it is not easy to save funds for the future when feeding a large family.

*“ [Who delivered the baby?] No, no, at home. There was my wife and some other woman at home…[How and why this decision was made?] I did not decide that. It was nighttime, the pain started at 9 pm as I told you, so there were no arrangements for cars. So, we thought if something happened, we would go in the morning. The time we prayed for Fajar (first of the daily five Muslim ritual prayer, performed from dawn to sunrise) and prepared to go (to the hospital), we were informed that the baby had been delivered. Or else, we were about to come to the hospital…[Have you planned earlier for the delivery like the transport cost and other costs?] No, how can we save money, 14 people eat (dependents), and all this money (for newborn’s admission) was borrowed from people. We did not know that we would face this hurdle”.* (Grandfather, Battagram, 18)

Pregnancy and childbirth were regarded as women’s business, and males were not usually involved in discussions until the last moment. Communication was delicate, based on each family structure.

*“[Who do you consult during pregnancy?] I didn’t tell anybody, not even my husband. When I need to go to hospital, then I tell my mother-in-law.*” (Mother, Battagram, 13)

### Theme 2: health literacy, influenced by family experiences

Regardless of educational background, the newborn care practice at home described by mothers and families were uniquely influenced by local customs and family experiences, but far from WHO recommendations in many ways.

#### a. Lack of early initiation and exclusive breastfeeding.

There was a rampant myth that breastmilk only comes out a few days after childbirth, with reluctance for early initiation. Mothers typically started breastfeeding two to four days after delivery. Interim feeding varied from formula milk, cow/goat milk, *kahwa* (tea), and infant drops. Mothers’ attitudes toward exclusive breastfeeding varied.

*“I give formula milk because my own is very little. One day I gave him kahwa (tea). It is good for chest issues, cough issues. The other day I gave cow’s milk mixed with water. One portion of milk, two portions of water.”* (Mother, Kohistan, 4)

*“On the first day mother goes through so much labor pain. On the second day I felt right then breastfed the baby. (On the first day) I gave goat’s milk, because if I had given her formula milk then she would be on it completely.”* (Mother, Mansehra, 9)

Distrust of her own breastmilk was narrated by one mother; this was further provoked by surrounding female family members.

*“Three of my daughters who died used to cry so much. People said it is because my milk is not good for their health. How I came to know this is by putting ants in my breastmilk. I put some ants in my breastmilk and the ants immediately died. My girls used to cry every day and they did not have any other disease, so I assumed that my milk was dangerous for their health. People also said that your milk has germs in it and the way it is not good for ants, similarly it is not good for your babies, either.”* (Mother, Mansehra, 6).

One staff member explained the vicious cycle wherein newborns started with bottle feeding, with soft bottle teats easier to suck than breast. It is therefore challenging for them to shift to direct breastfeeding, and they ultimately miss the colostrum.

*“Those (mothers) who feed without feeder (milk bottle) they give colostrum, those who use feeder they don’t (give colostrum). Human is lazy by nature. Babies don’t need to suck it from the feeder, so when they go on breastfeeding, the duration of three days would have passed already. Mothers are more concerned about their pain (than early breastfeeding initiation). I think they don’t even know the importance of their milk.”* (Staff, 23)

Discarding colostrum, and giving prelacteals to newborns, was also common. Mothers were typically advised by in-laws, but one mother changed behavior by learning from other mothers in the hospital.

*“For my boy (firstborn), on the second day I gave breastmilk. First day I didn’t have milk in my breast. First milk was greenish color, and I didn’t give that. I wasted away. Then I gave normal milk. The first time, my family members, sister-in-law, said to put out this milk and don’t give it to the baby. But this time, I gave it after 10 minutes. There were other women, who put their milk in the jug. So, I thought I should also put my milk in the jug. I am breastfeeding, I can’t buy formula milk.” (Mother, Battagram, 7)*

One grandfather mentioned that his daughter-in-law focused on breastfeeding according to the doctor’s advice, but he was not sure about colostrum.

*“She was in pain, so when she became conscious her elders told her to breastfeed the baby. Some said, give him kahwa (tea), and goat’s milk but we said no. Once a doctor told us that mothers’ milk is good… [The first milk that came to her breast after the delivery, was greenish in color?] I don’t know about it... I was told by a doctor that you people give cow’s milk to the babies for three days, don’t do it. Give mother’s milk immediately.”* (Grandfather, Battagram, 18)

*Ghutti*/*tahnik* (first sweet feed) is the Islamic ritual of rubbing the palate of a newborn baby with dates or honey, from the elderly, before breastmilk, so that the good attributes will be transferred to the baby. In Pakistan, there are two types of *ghutti* in relation to prelacteals; the other will be described later.

*“On the first day when we went home, we tried to give him anything. My mother-in-law gave him ajwa date (dates from holy Medina town, Saudi Arabia), and tea with a spoon but I think he didn’t take it. I don’t know about it, but they tried.”* (Mother, Mansehra, 14)

*Kahwa* (tea) is ordinarily administered for prevention and treatment of the common cold in the region for all generations. Alarming situations for infants were noticed, as milk bottles filled with *kahwa* (tea) were seen by some staff. The ingredients are often green tea and sugar, with or without some herbs/spices, boiled together. Some caregivers may use locally available poppy tea for infants as a local remedy for cough or crying; this was reported by several staff members as opioid overdose cases.

*“There is a conception if they give kahwa (tea) they will not get a respiratory infection. More they (babies) drink, more they lose weight. Muscles are wasted because they are not proteins. There is only sugar maintaining for brain. Doesn’t have any nutritional value. Sometimes they come back with too much kahwa (tea) and opioid poisoning. There is naloxone as antidote. We use them. We explain them the problem you are giving kahwa (tea) and this is causing him. He is floppy and not responding. Sometimes they understand, sometimes they don’t. We cannot interpret after they go home. I have seen twice opioid poisoning…Although parents didn’t tell their kahwa (tea) ingredients, adding opium seeds have been practiced since long, and (empiriacal) Naloxone treatment worked well.”* (Staff, 19)

Commercial probiotic products claiming effectiveness for infant abdominal pain are often used by families. This is also called *ghutti* (marketed infant drops) in Pakistan, but differs from the *ghutti* used in the traditional ritual of the first ever sweet feed for newborns, as illustrated above. One mother reported that it had also been used in the local hospital where she delivered the baby.

*“For the three days I had no breastmilk. Even though I was giving it to baby but there was no milk in it. For the three days the baby was eating ghutti (marketed infant drops). It’s liquid. Gave it to the baby in the hospital, too.”* (Mother, Mansehra, 12)

Calming down a crying baby seemed a priority to caregivers. As the case reported, the juice had been used for quick relief.

*“Patients came to the nursery, they were drinking juice and feeding it to the baby as well on the way to the hospital. They said they thought they should give it to the baby because he was crying.”* (Staff, 25)

#### b. Suboptimal bathing, wiping, wrapping, and umbilical cord care.

Hygiene/sanitation is another challenge in rural and remote areas. Many caregivers reported that they had not yet bathed the newborn. With temperatures dipping to sub-zero levels in winter in the area, caregivers were afraid to bathe newborns. Yet, newborns were not wiped daily either, which suggested that minimal hygiene was not maintained*.*

*“We haven’t given a bath yet (Day 22). We wiped after four days he was discharged (Day 8).”* (Mother, Battagram,13)

*“No, there was too much cold, so because of that fear, did not bathe him.”* (Grandfather, Battagram,18)

Different kinds of topical agent application for umbilical cord care were described by both mothers and staff. They included spirits, *Polyfax* (pharmaceutical brand antibacterial ointment), mustard oil, *ghee* (clarified butter), *kajal/kohl/surma* (blackish eye cosmetic), turmeric (spice), clay, and ash, to name a few. Whilst the effectiveness of folk remedies has mixed reviews, umbilical cord infection cases were noticed by staff.

*“I put kajal (blackish eye cosmetic) on umbilical cord, which was told by the elderly people.”* (mother, Battagram, 7)

*“Those who come to us from rural areas, have usually used kajal/kohl (blackish eye cosmetic) and turmeric (spice) on it. Those who delivered here only use spirit but those who come from outside use kajal/kohl (blackish eye cosmetic), especially who are delivered at home. When they go home and then come back, baby is all blackish. When we open the baby, it smells so bad, we see it’s infected. The majority have an omphalitis.”* (Staff, 21)

*“I noticed at home they use mustard oil, different other types of oil, and Dalda oil (refined oil brand). And by the way, I think healing gets fast with oils like Dalda and all. I myself have witnessed this in rural people. I have also seen they even use ashes. It works as antibiotic. It’s very rare but I have seen it with my own eyes.”* (Staff, 22)

*“At home people usually use mustard oil. I haven’t seen cords infected by the agent, but I have seen cords infected because it is covered with the diaper. If it is outside the diaper, then it is not infected. Some cases came upon admission, we have seen kohl (blackish eye cosmetic) and henna (herbal dye) used on their umbilical cords. Afghani people use henna on umbilical cords and genitalia, maybe because of its antibiotic properties.”* (Staff, 25)

The risk of hypothermia and the struggle for temperature control in neonates were a concern of many staff.

*“Their elders wear jackets, coats, and shawls but they bring baby naked. Without any protection and warm clothes. Sometimes they bring the baby in a single shawl of the mother. Baby should be warmed up and clothes properly.”* (Staff, 21)

*“Wrapping is OK, but they tie too much.”* (Staff, 20)

#### c. Influence of local/traditional family experiences.

Knowledge and practice of newborn care were strongly influenced by local, traditional, and family norms and experiences. Most newborns were not given the zero dose (birth dose) vaccination for polio and tuberculosis. Usually, parents need to take the newborn to the nearest vaccination center within a few days of birth; however, access to facilities and lack of consent from the elderly were obstacles.

*“Elders did not give it attention so I could not ask them to take the baby for injections.”* (Mother, Battagram, 7).

One staff member pointed out that, overall, newborn care was not updated from the parents’ generation, due to lack of information from outside the home and family hierarchy, or obedience to elders.

*“They are good in family support. But medically they are zero, to be honest…We don’t have access to social media awareness thing. Instead, we watch commercials of biscuits and chocolates and so on. We don’t see commercials which shows how to take care of babies and mothers. In India, there are many short films, and they are posting in facebook. In Pakistan, I didn’t see any of it…. If you bathe a child, he becomes sick. Baby is OK not to bathe 2-3 weeks. I think they are trying to follow from parents. They are not willing, but parents are willing.”* (Staff, 19)

The health literacy of the elders’ generation impacted decision making on whether to seek medical care. While one mother appreciated the absence of influence from elders at home, another mother explained that her mother-in-law would not agree to going to the hospital despite a medical emergency.

*“There is no elder person in our home, so we go to hospital.”* (Mother, Mansehra, 8)

*“Last time we could not come (to hospital for delivery) and even this time, we were not coming if I had not been bleeding at that time, delivery would have happened at home. My mother-in-law was saying it’s not your time when I was bleeding. She did not trust me that I am feeling labor pain. I said, take me to the hospital. Then, my aunt’s daughter-in-law said that she was bleeding so take her to the hospital immediately. Then they took me to the hospital.”* (Mother, Mansehra, 15)

### Theme 3: health seeking behavior, influenced by family circumstances

Multiple healing approaches are employed in resource limited areas, influenced by families’ socio-cultural and geographical circumstances. Some actions are harmful, while others are benign, but may delay the seeking of medical attention.

#### a. Home treatment by family knowledge and experience.

Some families attempted home treatment, such as direct exposure to sunlight for jaundice, as quoted previously in Theme1(b), and *kahwa* (tea) for chest infection and fits.

*“We brought him to hospital because he had a fever and was having some fits… We gave kahwa (tea) with sugar just a little, not much, because foam was coming from his mouth.”* (Father, Battagram,16)

#### b. Custom to seek *moluvi* (local Islamic preacher).

Other families took the sick newborn to the local *moluvi* (Islamic preacher), who they believed could heal patients religiously by giving or selling amulets.

*“Initially, he was fine, he had no issues and we went back to our area. Next day he turned yellow for which we did some medicine and Taweez (amulet) for him, and he became fine with that. Then he developed a chest infection. He was unable to cough easily. We took him to the private clinic and then to the government hospital. But no betterment was seen in him. After 22 days (from birth) we brought him here.”* (Mother, Kohistan, 1)

#### c. Distrust/quality of rural health facilities.

Often after failure of home and religious remedies, families sought medical care. Some attempted nearby clinics and were then referred or self-referred to bigger hospitals. Others traveled straight to the tertiary care hospital. In any case, underlying limited capacity and distrust of local rural health facilities were visible.

*“He was badly crying one night so took him to the nearby hospital but there were no doctors, so we came here.”* (Mother, Mansehra, 5)

*“They were saying leave him here (local hospital) and he will become okay. There was no proper management of oxygen. Even if the baby dies there, they will not tell you to take him back that is why babies get destroyed there.”* (Mother, Kohistan, 1)

### Theme 4: lack of preventive care

Theme 3(c) touched upon health service quality in rural health facilities, although lack of preventive care was revealed as an encompassing issue through all levels of healthcare, whether urban or rural, or private or government.

#### a. Short duration of postnatal stay in health facility.

Mothers were often discharged on the same day of normal vaginal delivery, and two to three days after cesarean section. Obviously, there is not enough time for postnatal observation, examination, health education, and counselling before discharging home.

*“After one hour from delivery I was discharged (from district headquarter hospital). No health education, PNC reminder was given to me. It (delivery)was nighttime and there was no doctor, only nurses were there.”* (Mother, Kohistan, 4)

#### b. No reminder of regular PNC and immunization.

Most mothers narrated that they did not return or were not even reminded to come back for regular PNC or routine immunization by healthcare providers, other than mothers coming for removal of sutures from a caesarean section.

*“(Did anyone tell you to come back for PNC?) No. I wanted to check up on the kid, but they said your baby is alright and there is no need for checkup.”* (Mother, Kohistan,1)

One mother returned for suture removal late due to financial constraints, but used the opportunity to report a newborn feeding issue. It implies that she did not have anyone or anywhere nearby to seek help for her newborn’s condition.

*“The doctor said, come to me after one week and I will unstitch your stitches. But I did not go to her for 10 days. We borrowed money and then went to her. I told her it’s been two days since my baby had not drunk milk.”* (Mother, Mansehra, 2)

#### c. Limited/ineffective health education/counselling.

There was a discrepancy regarding health education between service providers and recipients. Whilst staff indicated that they do provide health education, or at least try to, including that on breastfeeding, hygiene care, or family spacing, mothers and families rarely recalled health education or counseling during ANC or admission to health facilities.

*“[PNC] They said if you feel any issue then come for check-up but I was fine and my daughter was also fine, so I didn’t go… [Health education such as danger signs] The doctors didn’t tell me such things but the Lady Health Worker (community health worker) told me about these issues, and told me to consult a doctor if I face any such issues.”* (Mother, Mansehra, 8)

Staff members implied that mothers’ and families’ availability and time constraints hindered their ability to provide adequate health education.

*“Basically, we try to educate. You have to stimulate before you start pumping. I encourage them to breastfeed. There is a lactation nurse now. Whenever they come, I try to encourage. If you don’t give, child gets sick, infectious diseases. Obviously, they get burping, but it is normal. I try to clear all misconception but too many people so I can’t talk to them all. Mostly, they are fine when I talk, they agree to do it…. It is time issue. Although I have allocated time to communicate with all the parents, sometimes we call them on phone, phone is busy.”* (Staff, 19)

### Theme 5: high burden of LBW

The last theme that emerged was that the high LBW burden is an overarching familial, societal, and institutional challenge. Seven of the 18 newborns in the interviews were LBW or very low birth weight (VLBW). Social factors causing preterm birth or LBW, as well as insufficient LBW care in facilities, were revealed.

#### a. Maternal Malnutrition.

Among the 18 newborns’ mothers, eight experienced pregnancies five or more times, and among them five had given birth five or more times. The earliest age at marriage was 13, although remote women did not remember their exact age. Frequent pregnancies with short intervals and poor nutritional intake for pregnant and lactating women were demonstrated in our study.

One mother mentioned that she did not eat meat during her entire pregnancy, due to poverty and advice from religious preachers and doctors, but it is not clear who she referred to as doctors.

*“They say, doctors and moulvi (local Islamic preacher), said you shouldn’t eat chicken because you have germs in your body. For 10 months I ate only okra (lady finger). I am poor and I chose to be patient. I just wanted to have a baby.”* (Mother, Mansehra, 2)

The nutrition of mothers was not a priority for some in-laws, reflecting the lower hierarchy of daughters-in-law, as a staff member noted.

*“Some mothers-in-law come to postnatal ward and don’t take care of their daughter-in-law, neither give them breakfast on time, nor dinner or lunch, so we then tell them.”* (Staff, 24)

#### b. Poor understanding of complications.

Preterm birth can occur spontaneously, but one mother narrated that her preterm birth at eight months was triggered by intercourse. It was not clear if the husband knew or understood the doctor’s instruction.

*“First one was a girl who died after birth. It was preterm at seventh month. She was born alive, lived for six hours and then died. I didn’t have any idea in my first delivery, I literally had no idea at all. And for this delivery, I didn’t have any idea, but the doctor had prohibited us from having intercourse. But my husband at night (did it). On the same night the labor pain started. It was not with much force but (pain started after the intercourse).”* (Mother, Mansehra, 14)

#### c. LBW and VLBW babies cared for at home.

In our study, four newborns below 2 kg had been cared for at home (1.1 kg, 1.5 kg, 1.6 kg, and 1.7 kg). It was revealed that some birth facilities did not refer LBW babies to higher levels of care, and even those who were admitted to the newborn care unit could be discharged before recovery, due to bed control and the hospital environment for attendants.

*“I have twin babies, one boy and one girl. He was fine for 27 days. Yesterday, he vomited through his nose. The vomit was yellow in color. He was fed formula milk. Now doctors have taken out yellow water from his stomach. I don’t know the boy’s weight but maybe 1kg [medical record indicates 1.1kg].”* (Mother, Mansehra, 6)

*“Because of cross infection babies get sick. We tell them to take the baby from here. Those whom we don’t want to send back, they say we are taking the baby back. And those to whom we tell that the baby is better and take care of him at home, get infections here from other babies.”* (Staff, 21)

*“Smallest one was 0.9kg. I didn’t discharge him, but they wanted. Unfortunately, he died after three days, they told me. They were so tired. They stayed here more than a month. No place to feed, no place to stay. They are from Mansehra. They wanted to go home. I discharged with nasogastric tube so that he can take proper amount of feed, all the supplement, all the preterm probiotics, vitamins, as he was not taking orally. But still, he didn’t survive.”* (Staff, 19)

In contrast, one staff member shared a success story of a 0.9 kg VLBW baby who was discharged from the birth facility at less than 1 kg but was followed up closely on an outpatient basis.

*“One patient was about 0.9 kg weight…He was admitted with me for two or three days in the hospital. Then I counselled about care at home. He was regularly followed up with me (as an outpatient), I counselled about home care, what you will do, about complications, how to manage. And he survived. He is 6-7 years old…He survived without any disability, any complications. Preterm, LBW, many complications…VLBW babies, you have to work a lot, regular follow-up, regular follow-up. People are, attendants are uneducated. I mentioned every aspect in this case. I had a weekly baby check with attendants….Breastfeeding and feeding in this case.”* (Staff, 20)

## Discussion

This study illustrated the prevalent newborn home care practices, deeply influenced by local knowledge and experience, which could contribute to neonatal morbidity. It also highlighted other potential social and institutional contributors to poor neonatal outcome.

Among concerned care practices identified in this study, early and frequent breastfeeding and colostrum feeding should be promoted as a high priority, due to its cost-effectiveness and evidence-based nature; it is also less contradictory in socio-cultural contexts. Our participants expressed reluctance to breastfeed for a few days after childbirth because of the perceived normalcy of breastmilk insufficiency and the perceived neonatal illnesses, such as colic and crying; this corresponded with the findings of previous studies [18,19]. The myth of normalcy should be addressed as low milk production results from the infrequency of early breastfeeding [20]. The benefits of early and frequent breastfeeding should be communicated, including the prevention of excessive bleeding by accelerating postnatal uterus involution [21], transfer of mothers’ antibodies, providing warmth by skin-to-skin contact [22], and helping eliminate bilirubin to prevent physiological jaundice [23]. A small amount of colostrum, thick rich milk high in nutrients, is all a healthy term newborn needs during the early days [24]. This should be emphasized and supported by technical lactation support in an appropriate environment in all birth facilities.

Conversely, traditional beliefs about prelacteal feeding, such as *ghutti* (first sweet feed) and *kahwa* (tea), are deeply ingrained in local families, making behavior change difficult. *Kahwa* (tea), an aromatic green tea, or herbal tea, prepared by boiling ingredients such as cinnamon and cardamom, is used in India, Pakistan, and Afghanistan for its herbal healing properties [25]. However, toxic ingredients in *kahwa* (tea) such as poppy seeds are reported, and unfortunately, infantile opium poppy intoxication cases occur in the region [26]. Parents give infants poppy tea for excessive crying, cough, and fever, reflecting the local availability of poppy seeds, which are also used in food in rural Pakistan [26]. Even though traditional and complementary medicine has been important to prevent and manage chronic illness [27], precautions regarding remedies that might be hazardous for infants should be communicated. Honey, a dietary reservoir of *clostridium botulinum* spores, is a lethal toxin, causing infantile botulism cases in Pakistan and in immigrant families overseas [28–30]. The other kind of *ghutti* (marketed infant drops) in Pakistan is company made digestion liquids [31], often containing sweetener or honey and herbal laxatives, including *senna* and *cassia fistula* [32]. Since they are readily available in hospitals, affecting lactation establishment [33], the government should take measures to prevent easy access to prelacteals by implementing the Baby Friendly Hospital Initiative [34].

Similarly, umbilical cord care and hygiene frequency in resource-limited countries like Pakistan require local-specific consideration. Despite WHO’s basic principles of keeping the cord clean and dry [22], various topical agents are commonly applied to promote healing, hasten separation, and prevent pain, infection, and bleeding [35]. In rural Pakistan, putting *kajal/kohl/surma* (blackish eye cosmetic) on infants’ body and face is believed to prevent infection and evil eyes [9], but commercial products may contain potentially harmful lead and antimony. Basic cord care counseling, including handwashing, aseptic techniques, and avoiding potentially harmful substances is crucial, since cord site infection is one of the major causes of neonatal sepsis in Pakistan; the other is bottle feeding [36]. Having never bathed a term baby for weeks sounds unfamiliar in resource-rich countries, although WHO only recommends the first bath 24 hours after childbirth, leaving vernix on the skin for its antibacterial properties and temperature control [37]. Bathing frequency varies by country and climate [38], but delayed first baths and daily washing should be encouraged even in resource-limited settings [22,39]. Thermal control by appropriate dressing and wrapping is also essential to prevent hypothermia, respiratory distress, and malnutrition [40].

The study found a lack of preventive care in health service delivery in Pakistan, showing a discrepancy with statistics, and non-compliance with guidelines. The national statistics indicate that 64% of newborns and 69% of mothers attended PNC [3], whilst our participants were not reminded to return to health facilities for regular PNC. The Pakistan guideline indicates that discharge should be more than 12 hours after delivery, with a routine postnatal visit within the first week, documented in the home-based record [39]. In our study, discharge timing was much earlier and routine PNC or home-based records did not exist. Health education and counseling on newborn care and danger signs were rarely recalled by our respondents despite frequent ANC attendance and facility delivery. This was in line with the findings of previous studies [10,41]. Despite WHO prioritizing antenatal education to improve birth readiness and preparedness, no standard antenatal education is incorporated in routine checkups in Pakistan, and ANC service quality, especially counselling, is extremely poor [10,41–43]. In Pakistan, health promotion has been the area of expertise of the community Lady Health Workers, which made facility healthcare staff focus on curative medicine, untrained and unconcerned with preventive interventions. Interestingly in Pakistan, the number of doctors is double of that of nurses and midwifes [44]. The imbalance should be rectified as stipulated in the national strategy, since midwifery and nursing human resources are vital in antenatal education [44]. Health professionals originally from local communities speaking native language would be valuable asset for health promotion, especially in rural and remote area. A previous study indicated that family-oriented antenatal education, using a picture drama, by facility midwifes resulted in less pregnancy and neonatal complications [45]. Group sessions and pictorial home-based record booklets, known as the Maternal and Child Health Handbook, can also be used to enhance families’ birth preparedness [42,46]. Adequate time for health education in ANC, and before discharge after delivery, is crucial, and regular PNC along with zero dose vaccination should be promoted for optimal postnatal and newborn care.

Newborn home care for LBW and VLBW babies is even more challenging; nevertheless, early discharge occurred without caregiver readiness in our study. The guideline recommends referral to a hospital only if a baby is less than 1.5 kg and defines no specific cut off weight for LBW discharge, as long as a newborn gains weight for three consecutive days [22,39]. Kangaroo Mother Care (KMC), where mothers carry the baby skin-to-skin as an incubator, is recommended for a baby less than 2 kg [22,34,39], but the service is not available in many facilities in Pakistan, including our study hospital. Neonatal jaundice (NNJ) was a major cause of the admission in our study hospital, which was consistent with a study by secondary hospitals in Karachi, Sindh Province [47]. Majority could be managed by phototherapy without tertiary care requiring exchange transfusion [47], thus, appropriate referral by secondary service enhancement could reduce the burden of tertiary hospital. Providing comfortable environment to stay for admitted neonates’ attendants, especially for mothers, would prevent discharge against medical advice and promote breastmilk/breastfeeding.

Improving health service quality of care of small babies is imperative, and simultaneously, efforts to reduce preterm birth and/or LBW must be made. Among our six cases below 2.5 kg, four were preterm, one was from twins and one was full term but small for gestational age (SGA). Half mothers experienced more than five pregnancies, and the majority had past miscarriages. It is similar with a previous study in Peshawar, current KP Province, which determined presentation with anemia and the history of previous abortion/miscarriage as risk factors for SGA, in addition to teenage mothers, Afghan refugees and consanguinity [48]. Maternal nutrition and anemia, and potential triggers for preterm birth such as uncontrolled hypertension or intercourse during pregnancy, should be addressed in a culturally acceptable manner. The national guideline specifies antenatal anemia screening and counseling on nutrition, family planning and safer sex if at risk for infections [39], although health education was rarely reached to our participants. Conversely, good antenatal care reduces incidence of preterm babies, as stated in a study in Karachi [47].

**Fig 3:**
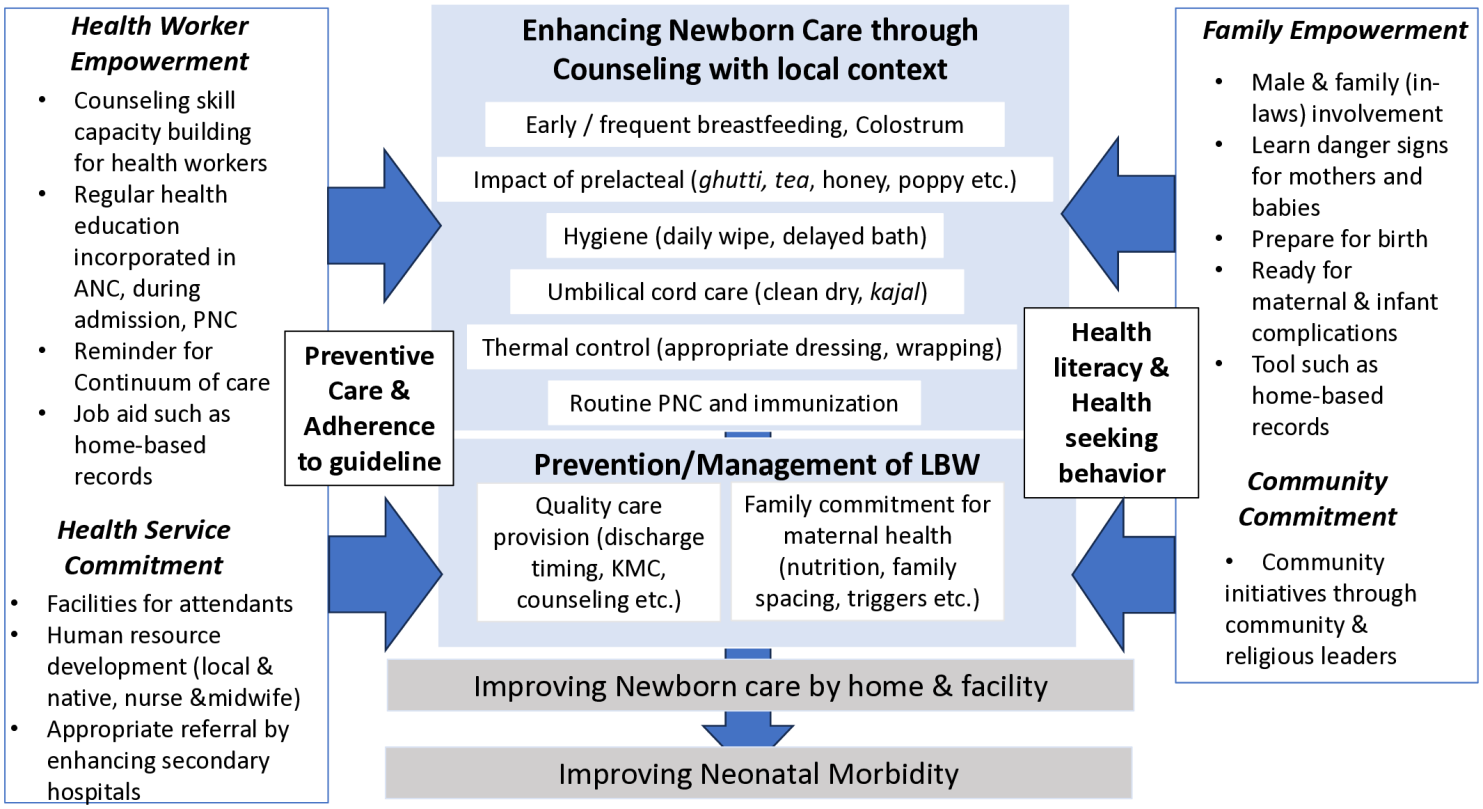
Way forward map.

The study found that cultural norms in South Asia, such as *purdah* (women seclusion), no male involvement in pregnancy and childbirth, and obedience to elders led to a lack of updated knowledge about newborn home care, and potential delays in seeking medical attention. Gender-based division of labor make females focus on marriage and childbearing, whilst many men are not involved or even unaware of obstetric and neonatal care needs as they are regarded as women’s business [49]. Due to less opportunity to interact with the outside world, women remain away from publicly exchanged ideas in their society [49], hence traditional home care remains unchanged from the elders’ generation. Within the Pakistani joint-family context, strengthening family health literacy is essential, rather than focusing on mothers only. Traditional families often prioritize honoring familial hierarchies, alternative medicine, religion, and resistance for forced compliance [50], making it crucial to understand and address these cultural norms. Encouraging male and family responsibility to protect maternal and child health through dialogues with community and religious leaders has been initiated by the local government and the development partner, and male involvement is gradually increasing in continuum of care [51]. Overall recommendations are summarized in the way forward map (Fig 3), nevertheless, some suggestions are a part of long-term strategies, given the structural, resource and cultural constrains. Thus, priority focus should be making small changes in everyday life and work, which can be practically manageable with current family structure and health human resources. The success story of the 0.9 kg VLBW baby narrated by the staff member, who was from the same tribe as the patient’s family, tells us that enhancing family health literacy through trust-based counseling can significantly improve infant survival. Incorporating culture-sensitive postnatal and newborn care education into facility daily practice would be the first step for sustainable reduction of neonatal morbidity.

### Strengths and limitations

The strength of this study is its geographical rarity, targeting mothers and families from rural areas, including Kohistan, Battagram, and Mansehra in Hazara division, KP province, Pakistan, where no previous research was conducted on the topic. Secondly, interviews with those who had just experienced newborn home care and subsequent newborn admission to the hospital enabled us to examine the potential causes and background of how newborn illness progressed, with less recall bias. Lastly, triangulation of sources and analysts; peer debriefing among various professionals, such as nurses, midwives, medical officers, and an anthropologist with clinical and research expertise in South Asian and other countries, were helpful to establish the credibility of the study, in addition to prolonged engagement and persistent observation by the first author.

However, it has limitations, including a population group bias since the study population does not represent the typical population, as they managed to travel to the tertiary hospital, potentially having better access to transport, finance, and families’ caring ability than the average in the region. We could only identify three mothers from Kohistan and no mothers from Torghar. To reach the unreached, further studies in Kohistan and Torghar with local interpreters may provide deeper context in more marginalized communities. Language barriers, and rural and remote mothers’ general inability to describe their stories and emotions, may have also impacted the findings. Open questions from the interview guide did not work easily for illiterate mothers. As we asked more probing questions, some potential answers might be restricted. Despite our interpreter’s fluency in Pashtun, Urdu and English, with a Pashtun cultural background, loss in interpretation could have occurred, especially for Kohistani mothers, double-interpreted from Kohistan to Urdu or Pashtun, and to English. However, each mother with a female relative ad-hoc interpreter provided us with honest and unpretentious answers and the research team also took ample time to cross-check the meaning, to minimize impact on data fidelity. Member check from mothers and families was impossible due to difficult follow-up once they left the unit. Nevertheless, we still believe that our study makes an important contribution to understanding postnatal and newborn care practices in these hard-to-reach areas in Hazara division, KP Province, Pakistan.

## Conclusion

This study identified lack of early initiation and exclusive breastfeeding; administration of prelacteals; and suboptimal bathing, wiping, wrapping, and umbilical cord care in newborn home care, which could make neonates susceptible to infection, dehydration, malnutrition, and hypothermia. Short postnatal stays, and lack of PNC and health education from health facilities left local mothers with only traditional care, influenced by familial traditional norms and gender role divisions. Multiple healing approaches and the service quality in local health facilities may have affected families’ ability to obtain timely medical attention. VLBW neonates had been cared for at home without caregivers’ readiness to nurse them. It is important to strengthen facility preventive care and families’ health literacy through culturally sensitive education in ANC, PNC, and during admission. Appropriate timing of discharge, with home care counselling with key family members, is also vital, considering the significant proportion of preterm and LBW infants.

## Supporting information

S1 Appendix Semi-structured interview guide. (DOCX)
