# Correction: Exploring postnatal and newborn home care experiences of rural and remote mothers in Pakistan whose neonates were admitted to the hospital: A qualitative study

**DOI:** 10.1371/journal.pone.0343875

**Published:** 2026-02-26

**Authors:** The PLOS One Staff

## Notice of Republication

This article was republished on February 9, 2026, to correct errors in the Supporting Information files. An incorrect version of S1 Appendix was published in error. The publisher apologises for this error.

## Supporting information

S1 File Originally published, uncorrected S1 Appendix. (DOCX)

S2 File Republished, corrected S1 Appendix. (DOCX)
