# Supplementary material for: Exploring postnatal and newborn home care experiences of rural and remote mothers in Pakistan whose neonates were admitted to the hospital: A qualitative study
Source: PLoS One. 2026 Jan 23;21(1):e0339957. doi: 10.1371/journal.pone.0339957 (PMC12829942; doi:10.1371/journal.pone.0339957)
Supplement: S1 Appendix — (DOCX) [file pone.0339957.s001.docx]

**Interview Guide (Mothers)**

**Exploring postnatal and newborn home care experiences by rural and remote mothers in Pakistan whose neonates were admitted in the hospital; A qualitative study**

**Introduction**

Researcher/Interpreter:

Researcher: My name is ( ), I am a researcher of this survey.

Interpreter: My name is ( ), and I am an interpreter.

Thank you very much for your participation in this study and interview.

- The purpose of this study is to learn your postnatal and newborn care experiences.

- The information we gather in this interview will be used to help us understand mothers’ efforts and challenges to care for babies in rural and remote area.

- We will write a report of what we learn from you and other people. We give recommendations to those who strategize healthcare services to make mothers’ childbearing and rearing more comfortable.

- Your name or other identifying information will not be used in the report.

- In order to understand your postnatal experiences, we would like to explore, starting from how you perceived and acted on your pregnancy and childbirth, then postnatal and newborn care, and how you found your baby sick and how you reached to this hospital.

- This is your story. There is no right or wrong answer to the questions, so please talk open and freely.

**Participation Request, Consent form, Withdrawal form**

-Would it be all right that we record our interview and take notes upon your approval?

-Could you please read through “Participation Request” (or interpreter read if the interviewee is illiterate), and when you understand the research details, could you please sign the consent form?

-Herewith, we also would like to give you a withdrawal form. If you feel uncomfortable that we use your story as data for the research, please sign the withdrawal form, and send it to the address in the Participation Request, or please contact us by email. We will not use your data in the research once we receive your withdrawal form.

(Collect signed form)

- Do you have any question before we start the interview?

**Demographic data**

- Could you please tell us your name?

- What language do you speak at home?

- Where do you live? How long does it take from home to here?

- What is your nearest health facility? Who do you consult when you are sick?

- How old are you? How old is your husband?

- How long have you been married?

- What is husband job, and your job if any?

- How many child(ren) do you have, and how many times have you been pregnant?

- How many family members do you live together, and who are they?

- Which grade did you complete in school? Which grade did your husband complete?

**Obstetric and newborn data**

- What is your baby’s name? How old is (s)he? What is baby’s health issue?

- Was it full-term delivery? What was the birth weight? What is the weight now?

- Where was the baby delivered? By whom was (s)he delivered?

- How many hours taken from labor pain to delivery? Did your baby cry immediately?

**Delivery/Childbirth**

- What were you doing on that day, and how did you know your childbirth is close?

- Which hospital (or home) did you go first, accompanied by whom? How was the decision made, and why?

- How was your baby delivered (normal vaginal delivery/C-section/Assisted delivery), and by whom (Doctor/nurse/LHV/Community Midwife/DAI)?

- What treatment/explanation did you receive, and how did you feel about the service provided to you?

- What kind of health education did you receive (breastfeeding, postnatal checkup, vaccination etc.)

- How was your experience and feeling during labor and childbirth?

**Post-natal and Newborn Care**

- When did you give breast milk for the first time? (within 1 hour of childbirth, on the delivery day, on the following day of delivery etc.) Why was it?

-Did you give colostrum (first breast milk with greenish color) to your baby? Please tell me what you know about first milk. (Where did you learn or who taught you?)

- Please tell me if gave the baby any food or drink apart from breastmilk. What was it? Who advised you?

- When did you give the baby a bath for the first time (how many hours or days after childbirth)? Who advised you?

- How do you take care of your baby’s umbilical cord?

-Was your baby infected for routine immunization? Why?

-Did you have any challenges about yourself and newborn after childbirth? If so, who do you consult with?

-Could you please tell us your overall feeling on experience to look after yourself and your newborn after childbirth?

**Referral/Transfer**

-How did you find your baby sick, and where did you take her/him first?

-How did you arrive at Ayub Teaching Hospital, accompanied by whom? Where do you stay now?

-How was the decision made, and why?

-How did you arrange the transport? How much did it cost, and how long did it take?

-What was your feeling when you need to go to tertiary care hospital?

-How do you understand your baby’s condition? How are you feeling now?

**Pregnancy**

-Could you please tell us about healthcare services (check-ups, ultrasound etc.) you utilized when you are pregnant? Or traditional medicine, prayer you attended, and why?

- When and where did you go (or you did not go) Antenatal Checkups? Who’ decision was it, and why did you (or your family) decide to go, and go to that facility?

- What kind of services do you remember you received (Ultrasound, Blood test, Urine Test several times, measuring abdomen with tape, weight, blood pressure etc.) ?

- What kind of health education/counselling do you remember you received? (discuss birth plan, danger signs, breast feeding etc.)

-How were you (or were you not) planning about your delivery, and why? Birth planning; decide and prepare in advance where to deliver, how to get there, how to save the required cost, who to accompany etc.

-Could you please tell us your daily routine/daily timetable when you are pregnant (time to wake up, chores, work, goat or buffalo care, meal time, time to sleep etc.). Is it same as when you are not pregnant?

-Could you please tell me about selfcare during pregnancy (or what you were mindful to do or not to do, or what you were told to do or not to do by family). For example, rest, nutrition, exercise, eating or drinking cold/hot or specific food?

- What is your typical breakfast, lunch, snack and dinner? How often do you eat meat/fish/beans?

**Strength and weakness**

- Did you have any physical/mental troubles about yourself during the whole course of pregnancy, delivery and newborn care?

- Are there any traditional or religious ceremony your family often conduct for pregnant woman or newborns?

- Could you please tell us who did you consult with, and trust the most during pregnancy, delivery and post-natal experience, and why (among your family, healthcare workers and others)?

-What do you evaluate the cooperation of your family members or healthcare workers? What did they do well and what they could have improved?

-What do you evaluate that you did well, and you could have improved during whole experience?

-Whilst other mothers or babies cannot reach health facility, what do you think is the difference you made to access to healthcare services?

Thank you for your time and valuable experience sharing.

If you have any question, please feel free to ask.
